# Respiratory Arrest in an Obese Pregnant Woman with Hyperemesis Gravidarum

**DOI:** 10.1155/2015/278391

**Published:** 2015-11-29

**Authors:** Ayumi Iwashita, Yosuke Baba, Rie Usui, Akihide Ohkuchi, Shigeaki Muto, Shigeki Matsubara

**Affiliations:** ^1^Department of Obstetrics and Gynecology, Jichi Medical University, Tochigi 09216, Japan; ^2^Department of Nephrology, Jichi Medical University, Tochigi 09216, Japan

## Abstract

A pregnant, non-Japanese-speaking Peruvian, and, thus, with communication difficulty, suffered hyperemesis gravidarum and had respiratory arrest, requiring cardiopulmonary resuscitation. The obese pregnant woman (prepregnancy weight: 107 kg) had vomited and lost 15 kg in bodyweight over appropriately 2 weeks prior to the arrest but had not complained due to communication difficulty, which, together with her obesity, prevented a Japanese obstetrician from noticing her severe condition. 1,000 mL of low potassium fluid plus thiamine was administered. She became unable to stand, suggesting lower-extremity-proximal-muscle weakness, and then respiratory arrest occurred. Hypopotassemia (2.3 mEq/L), pulseless electrical activity, and muscle weakness suggested the presence of severe potassium deficiency, which may have caused respiratory muscle paralysis, leading to the respiratory arrest. Hypercapnea was severer than expected for compensatory hypoventilation, indicating the presence of concomitant severe hypoventilation, which may also have contributed to respiratory arrest. She recovered with electrolyte and volume replacement. Respiratory arrest can occur with hyperemesis gravidarum, and obesity and communication difficulties can prevent the early detection of severe conditions.

## 1. Introduction

Hyperemesis gravidarum (HG) sometimes causes life-threatening conditions [1, 2], including Wernicke encephalopathy [3], renal failure [4], or liver dysfunction [5–7]. We report a pregnant woman with HG, in whom respiratory arrest occurred, which required cardiopulmonary resuscitation followed by mechanical ventilation. The patient was obese and a non-Japanese-language speaker (thus, with verbal communication difficulty), both of which may have prevented the early detection of severe conditions: marked weight loss and electrolyte imbalance accompanying HG. The mechanism of respiratory arrest will also be discussed.

## 2. Case Presentation

At 12^4/7^ weeks of gestation, a 34-year-old, 1x parous (normal vaginal delivery), non-Japanese-speaking Peruvian was admitted to this Japanese tertiary institute under cardiopulmonary resuscitation. She had no family or past histories of note. In 2014 May, at 9^4/7^ weeks, she visited a primary obstetric facility due to pregnancy, when her weight was 107 kg (height: 167 cm; body mass index: 38.3 kg/m^3^). One week later, at 10 weeks of gestation, she vomited and could not take food but she did not consult a doctor. Two weeks later (12^3/7^ weeks) she vomited approximately 20 times/day and could not stand up, and so she visited the primary facility. Her weight was 92 kg (15 kg weight loss over approximately 2 weeks), blood pressure was 92/78 mmHg, and pulse rate was 94 bpm. Intravenous volume replacement with 1,000 mL of solution (containing 4 mEq/L of potassium, 130 mEq/L of sodium, 5% glucose, and 100 mg of thiamine) was administered. In retrospect, she had complained little about her condition (frequent vomiting and difficulty standing) and the obstetrician failed to notice or paid insufficient attention to her weight loss and severe symptoms (especially frequent vomiting and difficulty standing). On the next day (12^4/7^), respiratory arrest developed, and she was transferred to this institute under cardiopulmonary resuscitation.

She was unconscious with blood pressure of 60/0 mmHg, and pulse of 120 bpm. As shown in Table 1, hemoconcentration, azotemia, and electrolyte abnormalities were marked: serum sodium: 122 mEq/L, chloride: 58 mEq/L, and potassium: 2.3 mEq/L. Arterial blood gas analysis under 5 L/min oxygen inhalation showed PO_2_: 201.9 mmHg, PCO_2_: 62.5 mmHg, HCO_3_^−^: 42.2 mEq/L, and pH: 7.447.

Data showed that the patient had mixed acid-base disturbances, namely, (1) chloride-sensitive metabolic alkalosis, (2) respiratory acidosis, and (3) high anion gap metabolic acidosis. Firstly, she had chloride-sensitive (hypochloremic) metabolic alkalosis: her urinary chloride concentration was very low (3 mEq/L) and plasma HCO_3_^−^ concentration was markedly elevated (42.2 mEq/L). This chloride-sensitive metabolic alkalosis may have been caused by protracted vomiting and insufficient fluid intake. Secondly, hypoventilation may have been severer than that expected to compensate for this metabolic alkalosis: the expected appropriate respiratory compensation can be calculated as PCO_2_ of up to 52.7 mmHg at most [8], but she had PCO_2_ of 62.5 mmHg, being much higher than that. This indicates that she had respiratory acidosis. Thirdly, she may have had high anion gap metabolic acidosis: the serum anion gap, calculated by Na^+^ − (Cl^−^ + HCO_3_^−^), was 19.8 mEq/L, being markedly higher than the normal value of 10 ± 2 mEq/L. Her serum concentrations of lactate and creatinine were increased to 7.4 mmol/L (normal: 0.5–2.0 mmol/L) and 7.94 mg/dL, respectively: the high anion gap acidosis may have been caused by lactic acidosis, which may have been due to hypovolemic shock, and uremic acidosis.

Renin-angiotensin-aldosterone (RAA) activity was markedly increased, possibly due to severe hypovolemia: the plasma renin activity and plasma aldosterone concentration were 41 ng/mL/hr (normal: 0.3–2.9 ng/mL/hr) and 1,310 pg/mL (normal: 35.7–240 pg/mL), respectively. This increased RAA activity may have aggravated hypopotassemia: her urinary potassium concentration (43 mEq/L) was high.

She became pulseless. Electrocardiography revealed ventricular tachycardia and an intermittent idioventricular rhythm but without cardiac output, indicating pulseless electrical activity. Brain computed tomography, which was performed soon after stabilization of her cardiac condition, revealed no abnormalities.

We continued cardiorespiratory resuscitation under mechanical ventilation with tracheal intubation. Cardiac output resumed after electrolyte replacement and dopamine administration. Her condition then rapidly resolved. Respiratory support was performed for 2 days and laboratory data had mostly normalized 3 days later (Table 1). Bedside neurological examination revealed no symptoms characteristic of Wernicke encephalopathy including ataxia, oculomotor disturbances, or delirium. On day 4 (13^0/7^ weeks), magnetic resonance imaging was performed, which revealed no abnormalities indicative of central pontine myelinolysis [1] or Wernicke encephalopathy, that is, intense abnormalities within the medial thalamus and mammillary bodies, or in the area surrounding the aqueduct and third ventricle [3]. On day 11 (14^0/7^ weeks) food intake became possible. Intravenous administration of electrolyte and fluid (adequate electrolytes, thiamine (100 mg/daily), and glucose) which we continued from her admission was stopped. On day 25 (16^0/7^ weeks), she was discharged. During the pregnancy course, neurological examination revealed no abnormal findings. Following a normal pregnancy course without sequelae, including neurological sequelae, she gave birth to a male infant weighing 3,036 g with a 5 min Apgar score of 9 at 40^5/7^ weeks. At six months postpartum, the patient and her baby were healthy. The baby showed a normal growth and normal development. Since some unfavorable outcomes have been reported in babies born from HG mother [2], the baby is under regular checkups of a pediatrician. Written informed consent for reporting was obtained from the patient.

## 3. Discussion

Two important clinical issues were suggested. HG can cause respiratory arrest, which requires mechanical ventilation. Physicians may overlook severe weight loss of an obese pregnant woman with HG, especially when difficulty in communication exists and when she complains little of her symptoms.

Firstly, HG can cause respiratory arrest. To our knowledge, there has been no report on respiratory arrest in HG. HG sometimes causes Wernicke encephalopathy due to vitamin B1 (thiamine) depletion. Theoretically, respiratory arrest can occur with HG-induced Wernicke encephalopathy. Its main lesion is considered to be mammillary bodies and structures surrounding the third ventricle, aqueduct, and fourth ventricle [3]. If the disease is very severe and prolonged thiamine deficiency exists, the myelin sheaths of central pons become more sensitive to changes in serum electrolyte levels (sodium, phosphate, and potassium) [1]: myelinolysis occurs there and can cause respiratory arrest. Although Wernicke encephalopathy cannot be completely ruled out in this patient, the following may reduce its possibility: (1) vitamin B1 had been intravenously administered before the onset of respiratory arrest; Wernicke encephalopathy usually does not deteriorate after vitamin B1 administration and (2) the patient showed complete recovery within a very short period, which contradicts the usual clinical course of Wernicke encephalopathy. Additionally, magnetic resonance imaging revealed no findings indicative of Wernicke encephalopathy or central pontine myelinolysis. Neurological examination also revealed no evident signs of Wernicke encephalopathy. Then, why did respiratory arrest occur?

We believe that severe potassium deficiency may have caused the respiratory arrest. Pre- or coexisting severe hypoventilation and severe obesity may also have been associated with it. A patient with HG frequently vomits gastric juice and, thus, the loss of hydrogen ions, sodium, chloride, and water in gastric contents leads to chloride-sensitive metabolic alkalosis and extracellular fluid (ECF) volume reduction. The present case exhibited both. Notably, this patient manifested severe ECF volume reduction: she lost 15 kg in bodyweight over 2 weeks, her systolic blood pressure was very low with tachycardia, and RAA activity was markedly elevated. This activated RAA system, in turn, increases the urinary excretion of potassium, resulting in hypopotassemia and potassium deficiency. Importantly, the potassium deficiency in this case was severer than expected from the level of hypopotassemia for the following two reasons: (1) she became unable to stand, strongly suggesting proximal muscle weakness of her lower limbs, which is characteristic of potassium deficiency and is typically observed in patients with periodical paralysis due to severe hypopotassemia [9] and (2) arrhythmia also strongly suggests that her potassium deficiency was marked. Physiologically, metabolic alkalosis can be compensated for by hypoventilation. However, in the present case, PCO_2_ was excessively elevated for the degree of hyperbicarbonatemia (HCO_3_^−^: 42.2 mEq/L). In short, this patient was much more hypoventilatory than expected for compensatory hypoventilation. Considering the coexistence of severe potassium deficiency and excessive hypoventilation, marked potassium deficiency may have caused respiratory muscle paralysis and thereby exacerbated hypoventilation, finally leading to respiratory arrest. Severe obesity may also have been associated with it. In fact, severe hypopotassemia, sometimes in the form of familial periodic paralysis, can manifest as muscle paralysis and ventilatory incapacity, similar to cases with various myopathies [9].

The second suggestion based on this patient's clinical course was that obesity may have prevented the early detection of severe weight loss. She lost 15 kg over 2 weeks; however, she did not complain of it, possibly due to (1) difficulty in communication and (2) her modest character (not too much complaining). The latter is subjective, but her attitude during hospitalization strongly suggested this character. Even though a woman may have both (1) and (2), obstetricians may have easily noticed severe conditions if a lean, or at least a moderately weighing, pregnant woman had lost that much weight. The obstetrician of the primary facility, having 20 years of experience, was well accustomed to dealing with HG. Regardless of this, the obstetrician overlooked her severe weight loss and, thus, only administered 1,000 mL of low potassium fluid. Although this is only based on speculation, if the doctor had suspected a severe status and checked serum electrolytes, then the severe electrolyte imbalance may have been detected, which may have naturally led this obstetrician to perform sufficient volume and potassium replacement. Then, she may have not developed respiratory arrest. Once again, her obesity, difficulty in communication, and modest character may have worked together unfavorably. According to the statistics of Japanese Ministry of Justice [10], foreign residents in Japan occupy approximately 1.6% (2,086,603/125,704,000) of Japanese population. Some of them have verbal communication difficulty. Countries other than Japan may have much higher foreign residents, and, thus, communication difficulty may be a common problem worldwide.

Data showed that women with HG with extreme weight loss had a variety of symptoms, including hypotension or muscle pain [2]. Electrolyte imbalance, especially hypopotassemia, is also well known. We believe that although this is the first report, to our knowledge, of HG patients with respiratory arrest, some near-miss cases may remain unreported. Patients with very severe HG also had neurological or psychological symptoms such as memory loss, confusion, or mood changes [2]. Some HG patients may become less communicative irrespective of the predisposing personality. We must note that, even without language problem, some patients with HG may not complain of their symptoms.

This patient survived without sequelae whereas some women with HG may die [1]. We believe that after noticing the respiratory arrest, our team treated this patient very quickly and adequately under cooperation with many specialists, that is, obstetricians, emergency medicine specialists, intensive care specialists, cardiologists, nephrologists, and radiologists. Such multidisciplinary team approach may have been effective for her complete recovery.

In conclusion, physicians must be aware that HG can cause respiratory arrest, requiring mechanical ventilation, and that weight loss, even though it is severe to the extent that it induces electrolyte imbalance, may be overlooked in an obese pregnant woman. This is especially true when difficulty in communication exists between a patient and caregivers.

## Figures and Tables

**Table 1:** Laboratory data during the course.

	Transfer	Day 3	Discharge (day 24)	Normal values	Units
Hb	14.8	9.3	10.5	(11.3–15.2)	g/dL
Ht	42.8	27.6	30.6	(33.4–44.9)	%
BUN	89	38	4	(8–20)	mg/dL
Cr	7.94	1.46	0.4	(0.38–0.90)	mg/dL
Na	122	146	139	(136–148)	mEq/L
K	2.3	4.1	3.6	(3.6–5.0)	mEq/L
Cl	58	107	106	(96–108)	mEq/L
Albumin	3.7	2.2	3	(3.9–5.1)	g/dL
T-bil	1.28	0.75	0.47	(0.40–1.50)	mg/dL
Vit. B1	912	—	—	(24–66)	ng/mL
pH^*∗*^	7.447	—	—		
PCO_2_^*∗*^	62.5	—	—	(35–45)	mmHg
HCO_3_^−^^*∗*^	42.2	—	—	(24–26)	mEq/L
Lactate acid^*∗*^	7.4	—	—	(0.5–2.0)	mmol/L

Hb: hemoglobin, Ht: haematocrit, and BUN: blood urea nitrogen.

Cr: serum creatinine, Na: sodium, and K: potassium.

Cl: chlorine, T-bil: total bilirubin, and vit. B1: vitamin B1.

HCO_3_^−^: bicarbonate ion.

^*∗*^Arterial blood gas analysis.

## Abbreviations

ECF: Extracellular fluid
HG: Hyperemesis gravidarum
RAA: Renin-angiotensin-aldosterone.
